# Fibroblast growth factor 21 (FGF21) promoter methylation drives the progression of chronic hepatitis B by mediating oxidative stress and inflammatory immunity

**DOI:** 10.1128/spectrum.02769-25

**Published:** 2025-11-19

**Authors:** Xue Li, Feng Zhang, Hanxu Zhu, Zhezhe Tian, Miaomiao Xu, YuChen Fan, Shuai Gao, Kai Wang

**Affiliations:** 1 Department of Hepatology, Qilu Hospital of Shandong University 91623, Jinan, China; 2 Institute of Hepatology, Shandong University 12589, Jinan, China; American University of Beirut, Beirut, Lebanon

**Keywords:** FGF21, HBV, oxidative stress, immune inflammation, methylation

## Abstract

Hepatitis B virus (HBV) infection can cause liver damage through oxidative stress (OS) and immune-inflammatory responses. This study aims to explore the clinical significance of fibroblast growth factor 21 (FGF21) in the development and progression of chronic hepatitis B (CHB). A total of 336 participants were recruited, including 320 CHB patients and 16 healthy controls. The expression of FGF21, immune cytokines, and OS-related molecules in peripheral blood mononuclear cells (PBMCs) was detected using real-time quantitative polymerase chain reaction. The methylation level of the FGF21 gene promoter in PBMCs was detected using TaqMan probe-based quantitative methylation-specific PCR. The expression level of FGF21 in the peripheral blood of CHB patients was higher than that of HC, but the methylation level of the FGF21 promoter was lower than that of HC, especially in patients during the immune activation phase. The mRNA expression levels of CXCR3 and CCL5 in PBMCs of CHB patients during the immune activation and reactivation phases were higher than those in other clinical stages. Single-cell analysis revealed that CXCR3 and CCL5 expression in the immune tolerance and immune activation phases with high HBsAg expression was closely related to T lymphocytes (T cells) and natural killer cells (NK cells) and was highly expressed in CD4 and CD8 T cells and NK cells. In addition, the mRNA expression levels of Nrf2 and GPX4 in the reactivation phase were higher than those in other clinical stages. The mRNA expression level and methylation level of FGF21 in PBMCs of CHB patients were correlated with the viral load, immune inflammation, and OS levels during the antiviral treatment course of CHB. The methylation level of the FGF21 promoter has the potential to become a non-invasive biomarker for monitoring the progress of antiviral treatment in CHB.

**IMPORTANCE:**

This study conducted an in-depth exploration of the application of methylation detection technology, analyzing its value and driving mechanism in the oxidative stress and immune-inflammatory balance during the course of chronic hepatitis B. The study analyzed the methylation patterns of the FGF21 promoter and the expression levels of its receptor FGFR1, as well as the expression levels of chemokines CXCR3, CCL5, and oxidative stress factors GPX4 and Nrf2 in the immune tolerance period, immune clearance period, immune control period, and reactivation period of chronic hepatitis B. It clarified the association between these molecules and the FGF21/FGFR1 axis and revealed the synergistic or antagonistic mechanisms of these molecules in the oxidative stress and inflammatory vicious cycle. At the same time, this study also explored the value of FGF21 promoter methylation in disease diagnosis and prognosis, providing a theoretical basis for evaluating the antiviral treatment effect and disease progression of chronic hepatitis B.

## INTRODUCTION

Chronic hepatitis B virus (HBV) infection remains a major global health challenge. The progression and outcomes of chronic hepatitis B (CHB) are modulated by dynamic interactions among viral factors, host immune responses, and environmental conditions. Persistent HBV infection can induce T-cell activation, promote immune senescence, and lead to apoptosis-mediated impairment of virus-specific T-cell responses (1, 2). The disease progresses through four distinct phases: immune tolerance (IT), immune activation (IA), low replication (LR), and reactivation (RA) (3, 4). Each phase exhibits pathological features closely linked to alterations in the immune microenvironment, with oxidative stress (OS) and inflammatory responses serving as core mechanisms underlying liver injury and fibrogenesis. In clinical practice, viral load—measured via HBV-DNA, HBsAg, and HBeAg—is a critical marker for assessing antiviral treatment response and replication activity. However, it fails to fully capture the efficacy of antiviral therapy or the intrahepatic immune status (5, 6). Thus, integrating biomarkers of immune inflammation and OS-related cytokines provides a more comprehensive reflection of intrahepatic immune responses.

Fibroblast growth factor 21 (FGF21), a member of the fibroblast growth factor family, functions as a metabolic stress hormone. As a key regulator of hepatic metabolic homeostasis, FGF21 plays a vital role in protecting organs from damage. Previous studies (7, 8) have identified FGF21 as a novel biomarker with significant diagnostic and prognostic value in cardiovascular diseases, diabetes, and non-alcoholic fatty liver disease (NAFLD). FGF21 exerts anti-inflammatory effects by enhancing Nrf2-mediated antioxidant capacity and suppressing the NF-κB signaling pathway, thereby mitigating inflammation, cell death, and hypertrophy independent of dyslipidemia (9). Emerging evidence suggests that fibroblast growth factors (FGFs) mediate organ crosstalk by binding to specific FGF receptors (FGFRs) and their co-receptors, regulating intracellular responses under metabolic stress (10). Nuclear factor erythroid-2-related factor 2 (Nrf2) is a transcriptional master regulator of cytoprotective genes, including antioxidants and glutathione-synthesizing enzymes. One study (11) reported that recombinant FGF21 (rFGF21) activates FGFR1, increasing its binding to Kelch ECH-associated protein 1 (Keap1), which disrupts Keap1-Nrf2 interaction and promotes Nrf2 release. Additionally, Cheng et al. (9) demonstrated that glutathione peroxidase 4 (GPX4), a downstream target of Nrf2, participates in ferroptosis regulation. Another investigation (12) suggested that FGF21 protects against lung ischemia-reperfusion injury by inhibiting ER stress-induced apoptosis via the FGFR1/PPARδ pathway.

In addition to the role of FGF21 in oxidative stress mentioned above, the value of FGF21 in immune inflammation has also attracted attention. Wang et al. (13) further demonstrated that FGF21 is released by phagocytic leukocytes and mediates autocrine regulation of innate immune functions. Inflammatory infiltrates in injured livers comprise diverse immune cell subsets, including macrophages, dendritic cells, T cells, NKT cells, and B cells. Recent studies have also highlighted the role of chemokines in liver diseases. Cytokines and chemokines are crucial in initiating, sustaining, and regulating immune homeostasis and inflammatory processes (14). C-C motif chemokine ligand 5 (CCL5), also known as RANTES, plays a significant role in leukocyte chemotaxis and activation (15). In CHB patients, serum CCL5 levels are markedly elevated in those with moderate to severe liver disease compared to mild cases, indicating that viral infection activates innate and adaptive immune systems early on, triggering CCL5 production from T lymphocytes, macrophages, platelets, and other immune cells (16, 17). Beyond CCL5, C-X-C motif chemokine receptor 3 (CXCR3) and its ligands are key drivers of inflammation, with expression levels correlating with inflammatory severity (18). Clinical and animal model studies indicate that CXCR3 and its ligands recruit immune cells to hepatic inflammatory sites. CXCR3 also influences α-naphthylisothiocyanate (ANIT)- and triptolide-induced bile acid (BA) metabolic dysregulation and immune cell recruitment. It contributes to ER stress in ANIT-induced cholestatic liver injury (CLI) and oxidative stress in triptolide-induced CLI. Correlation analyses have further linked CXCR3-induced BA accumulation to cytokines secreted by immune cells and the initiation of ER/OS pathways (19). Throughout disease progression, OS and immune responses can induce changes in DNA methylation status.

Currently, the relationship between immune inflammation and OS factors in CHB progression remains poorly understood. The expression patterns and interaction networks of these molecules across different CHB phases, particularly during immune activation and reactivation, are yet to be fully elucidated. This study aims to analyze the expression and clinical significance of FGF21 promoter methylation and its receptor FGFR1, along with the chemokines CXCR3 and CCL5, and OS factors GPX4 and Nrf2, during the immune tolerance, clearance, control, and reactivation phases of CHB. It seeks to clarify the correlation between these molecules and the FGF21/FGFR1 axis, uncovering their synergistic or antagonistic mechanisms in the vicious cycle of OS and inflammation. Furthermore, it explores the diagnostic and prognostic value of FGF21 promoter methylation, providing a theoretical basis for evaluating antiviral therapy efficacy and disease progression in CHB.

## MATERIALS AND METHODS

### Patients

This study recruited 16 healthy controls (HC) and 320 CHB patients who visited Qilu Hospital of Shandong University from May 2024 to May 2025. All patients with CHB were selected and clinically staged into the IT phase, IA phase, LR phase, and RA phase based on 2018 AASLD Practice Guidelines (4, 20). This study was reviewed and approved by the Medical Ethics Committee of Qilu Hospital of Shandong University (KYLL-202306-021-1) and conducted in accordance with the Declaration of Helsinki. All participants signed informed consent forms after understanding the experimental process and the required specimens.

### PBMC isolation and DNA and RNA extraction

Peripheral blood mononuclear cells (PBMCs) were isolated from blood using the Ficoll-Plaque Plus gradient centrifugation method. Genomic DNA was extracted from PBMCs using TRIzol reagent (Invitrogen, Carlsbad, CA, USA). Total RNA was extracted from PBMCs using TRIzol reagent (Invitrogen, Carlsbad, CA, USA), and cDNA was synthesized using a cDNA synthesis kit (Fermentas, Vilnius, Lithuania) according to the manufacturer’s instructions. The primer sequences for measuring the mRNA expression levels of FGF21, FGFR1, CXCR3, CCL5, Nrf2, and GPX4, as well as β-actin, and the probe sequences for FGF21 and β-actin are listed in Table 1. The relative expression of FGF21, FGFR1, CXCR3, CCL5, Nrf2, and GPX was calculated using the 2^−ΔΔCT^.

**TABLE 1:** Sequences of the used primers and probes

Gene	Forward primer sequence (5′−3′)	Primer/probe sequence (5′−3′）
RT-qPCR		
FGF21	CTGCAGCTGAAAGCCTTGAAGC	GTATCCGTCCTCAAGAAGCAGC
FGFR1	GCACATCCAGTGGCTAAAGCAC	AGCACCTCCATCTCTTTGTCGG
CXCR3	ACGAGAGTGACTCGTGCTGTAC	GCAGAAAGAGGAGGCTGTAGAG
CCL5	CCTGCTGCTTTGCCTACATTGC	ACACACTTGGCGGTTCTTTCGG
Nrf2	CACATCCAGTCAGAAACCAGTGG	GGAATGTCTGCGCCAAAAGCTG
GPX4	ACAAGAACGGCTGCGTGGTGAA	GCCACACACTTGTGGAGCTAGA
ACTB	ATGGGTCAGAAGGATTCCTATGTG	CTTCATGAGGTAGTCAGTCAGGTC
Methylight		
FGF21	TTATTAAGACGTAGAGATCGGTAGT	TCACGTAACTTACTTAACCTTATCAAT
ACTB	TGGTGATGGAGGAGGTTTAGTAAGT	AACCAATAAAACCTACTCCTCCCTTAAA
Probe oligo sequence		
FGF21	AACGACTCACCCTCCTTATCCTACCC	
ACTB	ACCACCACCCAACACACAATAACAAACACA	

### DNA bisulfite modification

Extracted DNA was subjected to bisulfite modification using the EZ DNA Methylation Gold Kit (Zymo Research, Orange, CA, USA). MethyLight was performed using the EpiTect MethyLight PCR + ROX Vial Kit (QIAGEN, Hilden, Germany). Thermal cycling conditions included 95°C for 15 min, followed by 50 cycles of 95°C for 15 s and 60°C for 1 min. The final methylation result was expressed as PMR. PMR = 100% × 2 ^exp. [Delta Ct(target gene-control gene) Sample - Delta Ct (target gene-control gene) M.SssI-Reference]^ (21).

### Single-cell analysis

From the GSE247322 data set in the Gene Expression Omnibus (GEO) database, we obtained single-cell RNA sequencing (scRNA-seq) data from five CHB patients with high HBsAg levels. We used the R version 4.2.2 and Seurat version 4.3.0 software packages for further analysis. The data files were merged, log-normalized, and data integration was applied using reciprocal principal component analysis (k.anchor = 20, dims = 50) to correct for batch effects. We then selected 2,000 highly variable genes for principal component analysis (PCA) dimensionality reduction. The optimal principal components (PCs) were chosen for cell clustering analysis, followed by manual annotation. Finally, the expression of CXCR3 and CCL5 was visualized using “FeaturePlot.”

### Statistical analyses

R (version 4.2.2), GraphPad Prism (version 10), and SPSS (version 27) were used for statistical analysis. Quantitative variables were expressed as median (25th percentile; 75th percentile). Categorical variables were expressed as numbers (%). The Kruskal-Wallis test and Dunn’s test were used to compare quantitative variables. When making comparisons between groups, we use the letter system for labeling. If there are identical letters, it indicates no significant difference among the groups. If the letters are different, it means there is a significant difference between the groups. The chi-square test was used to compare categorical variables. Spearman’s rank correlation test was used to analyze the relationship between PMR and various indicators. A corrected *P* < 0.05 was considered statistically significant.

## RESULTS

### Patient screening and basic clinical information

**Fig 1:**
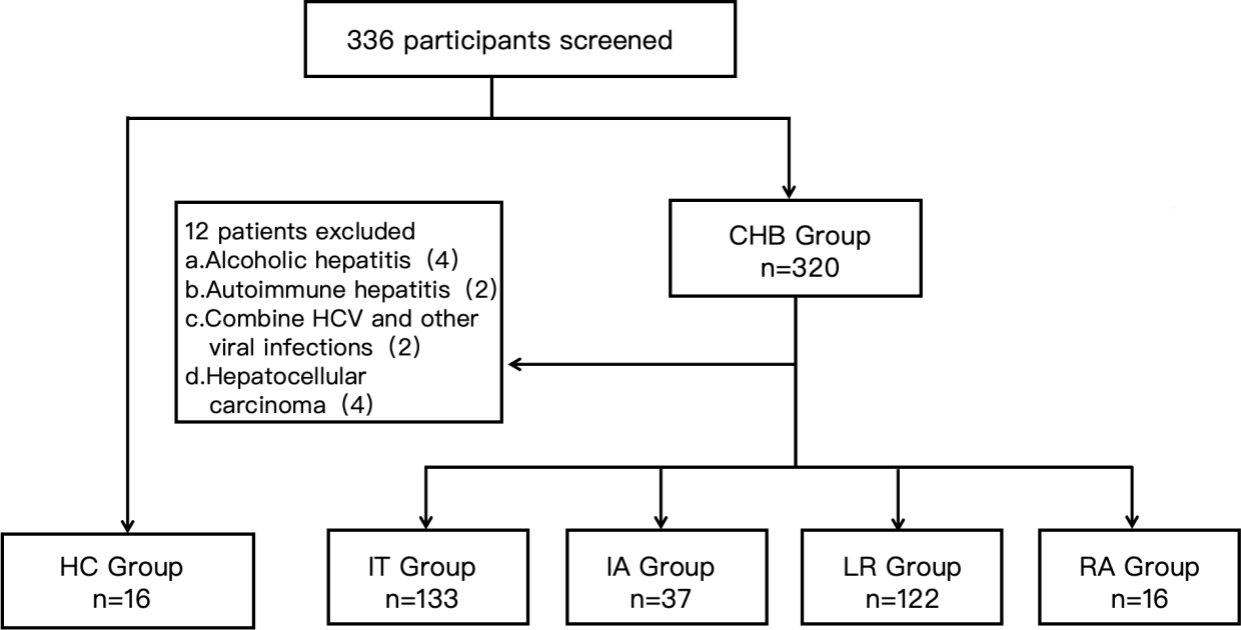
Exclusion criteria for participants.

Figure 1 illustrates the patient screening process and exclusion criteria for the experiment. In this study, a total of 336 subjects were recruited, including 16 HCs and 320 CHB patients, among whom 4 had alcoholic liver disease, 2 had autoimmune hepatitis, 2 had other types of hepatitis, and 4 were excluded due to hepatocellular carcinoma (HCC). Ultimately, 308 CHB patients were included. Table 2 presents the basic clinical data of the subjects.

**TABLE 2:** General clinical characteristics of the patients^*c*^^,^^*d*^

Parameter	IT (*n* = 133)	IA (*n* = 37)	LR (*n* = 122)	RA (*n* = 16)	HC (*n* = 16)	*P* value
Male, *n* (%)	105 (78.95)	32 (86.49)	74 (60.66)	11 (68.75)	5 (31.25)	0.001^*b*^
Age (years)	41.00 (35.00, 51.00)	41.00 (36.00, 45.50)	46.00 (38.00, 54.25)	48.00 (39.00, 51.75)	41.00 (23.50, 50.50)	0.0128^*a*^
log10 [HBV-DNA]	5.53 (4.65, 6.53)	4.37 (3.64,5.65)	3.52 (2.72, 4.38)	6.20 (5.51, 6.52)	NA^*e*^	<0.001^*a*^
HBsAg (IU/mL)	7,719.20 (4,246.95, 19,999.41)	6,742.51 (2,714.45, 23,058.22)	1,707.35 (746.98, 4,192.97)	1,479.57 (662.68, 2,410.54)	NA	<0.001^*a*^
HBeAg (IU/mL)	126.82 (2.86, 1,123.31)	8.70 (2.47, 146.03)	0.39 (0.36, 0.45)	0.41 (0.38, 0.47)	NA	<0.001^*a*^
ALT (U/L)	24.00 (19.00, 30.50)	56.00 (44.00, 77.00)	22.00 (16.00, 27.00)	54.50 (43.75, 69.25)	14.50 (10.50, 23.75)	<0.001^*a*^
AST (U/L)	21.00 (18.00, 25.00)	46.00 (41.50, 50.50)	22.00 (16.00, 27.00)	54.50 (43.75, 69.25)	18.50 (15.25, 20.00)	<0.001^*a*^
TBIL (µmol/L)	12.90 (9.90, 18.50)	14.80 (12.00, 22.30)	13.90 (9.60, 18.03)	11.05 (9.65, 18.65)	10.80 (7.80, 16.70)	0.220^*a*^
ALB (g/L)	47.00 (45.30, 49.00)	47.70 (45.80, 50.05)	46.40 (44.58, 48.20)	48.25 (44.85, 49.13)	44.90 (42.60, 48.10)	0.034^*a*^
AFP (ng/mL)	2.74 (1.95, 3.79)	4.00 (2.47, 5.48)	2.47 (1.84, 4.00)	5.27 (2.60, 8.76)	2.24 (1.11, 3.65)	0.013^*a*^

^*a*^ Kruskal–Wallis H test.

^*b*^ Chi-square test.

^*c*^ Quantitative variables were expressed as medians (25th, 75th percentage).

^*d*^ Qualitative variables were expressed as number (percentage).

^*e*^ NA, not available.

### FGF21 and its receptor FGFR1 mRNA expression and FGF21 promoter methylation levels

**Fig 2:**
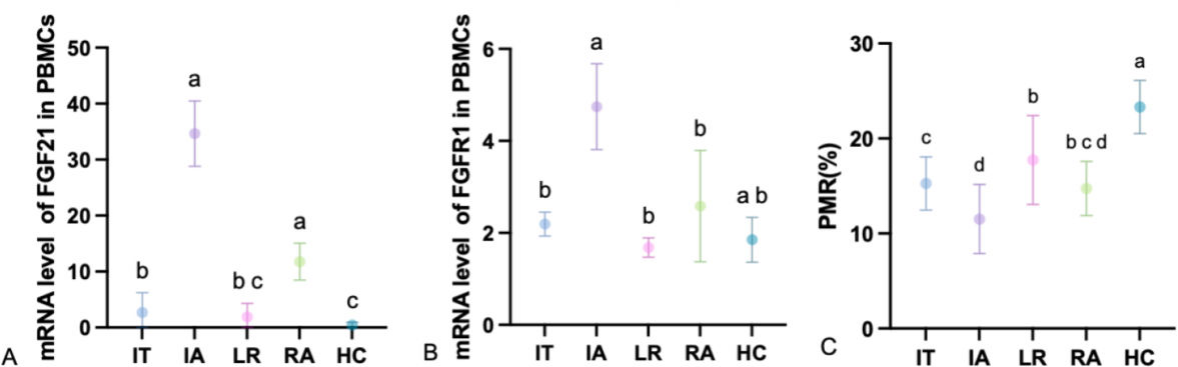
FGF21, FGFR1mRNA expression, and FGF21 PMR level. (**A**) Levels of FGF21 mRNA in IT, IA, LR, RA, and HC. (**B**) Levels of FGFR1 mRNA in IT, IA, LR, RA, and HC. (**C**) FGF21 PMR levels in PBMCs from IT, IA, LR, RA, and HC. Use “abc” to indicate whether there is a significant difference in comparisons between groups. If the letters for each group are different, it indicates that there is a significant difference between the groups (*P* < 0.05). If the letters are the same, it means that no significant difference was observed in the comparisons between the groups.

This study employed the MethyLight method to quantitatively detect the mRNA expression of FGF21 and its receptor FGFR1, as well as the FGF21 promoter methylation levels in HC and CHB patients, with PMR values calculated accordingly. The levels of FGF21 mRNA in IT, IA, LR, RA, and HC are shown in Fig. 2A. The relative mRNA level of FGF21 was significantly lower in IT (*P* < 0.0001), LR (*P* < 0.0001), and HC (*P* < 0.0001) compared to IA, and lower in IT (*P* < 0.0001), LR (*P* < 0.0001), and HC (*P* < 0.0001) compared to RA. The levels of FGFR1 mRNA in each group are shown in Fig. 2B, with the relative mRNA level of FGFR1 being significantly lower in IT (*P* = 0.0023), LR (*P* = 0.0007), and RA (*P* = 0.0452) compared to IA. The FGF21 promoter methylation levels in each group are represented by PMR, as shown in Fig. 2C, with FGF21 PMR levels being significantly higher in IT (*P* = 0.0006), LR (*P* < 0.0001), and HC (*P* < 0.0001) compared to IA, and higher in LR (*P* = 0.0002) and HC (*P* < 0.0001) compared to IT.

### Differential expression levels of immune inflammatory and oxidative stress factors in patients with HC and CHB at different stages

**Fig 3:**
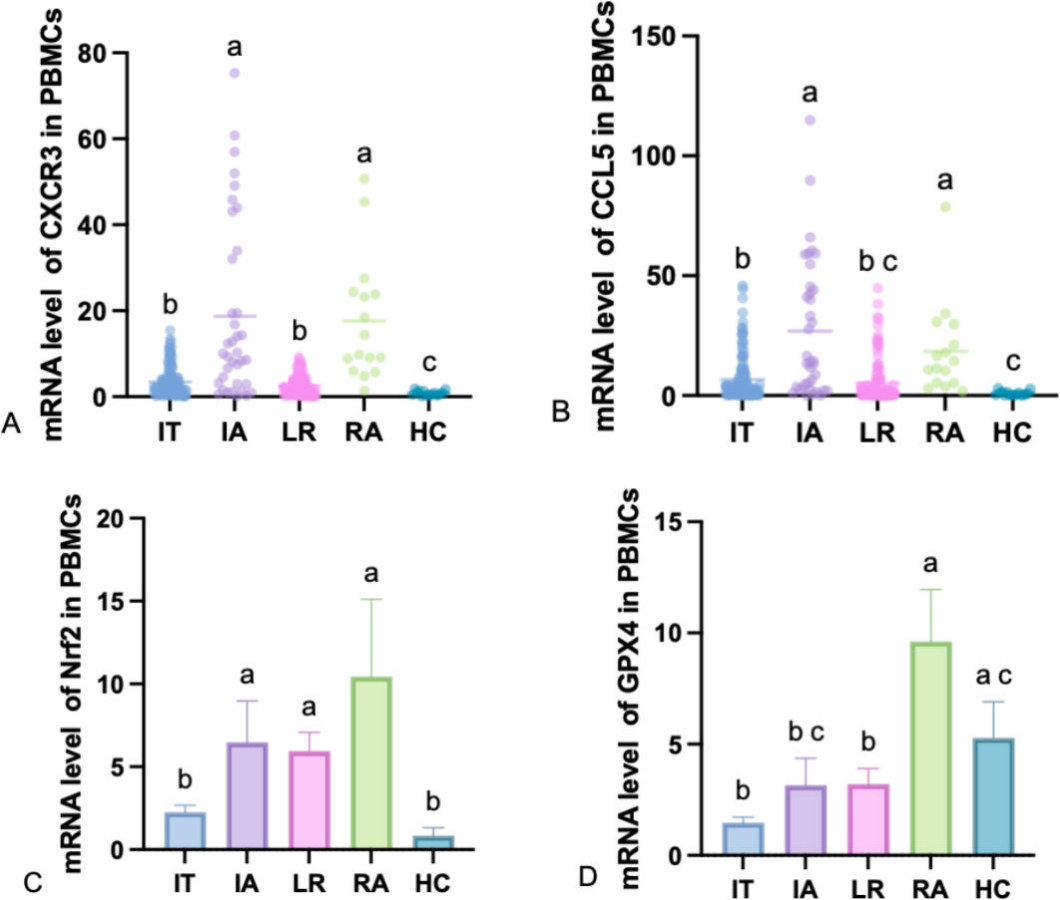
The expression patterns of CXCR3, CCL5, Nrf2, and GPX4 in different groups. (**A**) Levels of CXCR3 mRNA in IT, IA, LR, RA, and HC. (**B**) Levels of CCL5 mRNA in IT, IA, LR, RA and HC. (**C**) Levels of Nrf2 mRNA in IT, IA, LR, RA and HC. (**D**) Levels of GPX4 mRNA in IT, IA, LR, RA, and HC. Use “abc” to indicate whether there is a significant difference in comparisons between groups. If the letters for each group are different, it indicates that there is a significant difference between the groups (*P* < 0.05). If the letters are the same, it means that no significant difference was observed in the comparisons between the groups.

As shown in Fig. 3, we further examined the expression levels of the immune inflammatory markers CXCR3 and CCL5 mRNA, as well as the OS markers Nrf2 and GPX4 mRNA, in PBMCs of HC and patients with CHB at different stages. The results showed that the relative mRNA level of CXCR3 was significantly lower in IT (*P* < 0.0001), LR (*P* < 0.0001), and HC (*P* < 0.0001) than in IA, and lower in IT (*P* < 0.0001), LR (*P* < 0.0001), and HC (*P* < 0.0001) than in RA (Fig. 3A). The relative mRNA level of CCL5 was significantly lower in IT (*P* = 0.0003), LR (*P* < 0.0001), and HC (*P* < 0.0001) than in IA and lower in IT (*P* = 0.0081), LR (*P* < 0.0001), and HC (*P* < 0.0001) than in RA (Fig. 3B). It is worth noting that the IA group showed the highest mean expression, though the data distribution suggests potential heterogeneity within this patient phase, with a subset of individuals driving the elevated mean. The relative mRNA level of Nrf2 was significantly lower in IT (*P* = 0.0377) and HC (*P* = 0.0031) than in IA and lower in IT (*P* = 0.0012) and HC (*P* = 0.0001) than in RA (Fig. 3C). The relative mRNA level of GPX4 was significantly lower in IT (*P* < 0.0001), IA (*P* = 0.0016), and LR (*P* < 0.0001) than in RA and lower in IT (*P* = 0.0001) and LR (*P* = 0.0139) than in HC (Fig. 3D).

### Upregulation of CXCR3 and CCL5 in natural killer cells and T cells in CHB patients with high HBsAg

**Fig 4:**
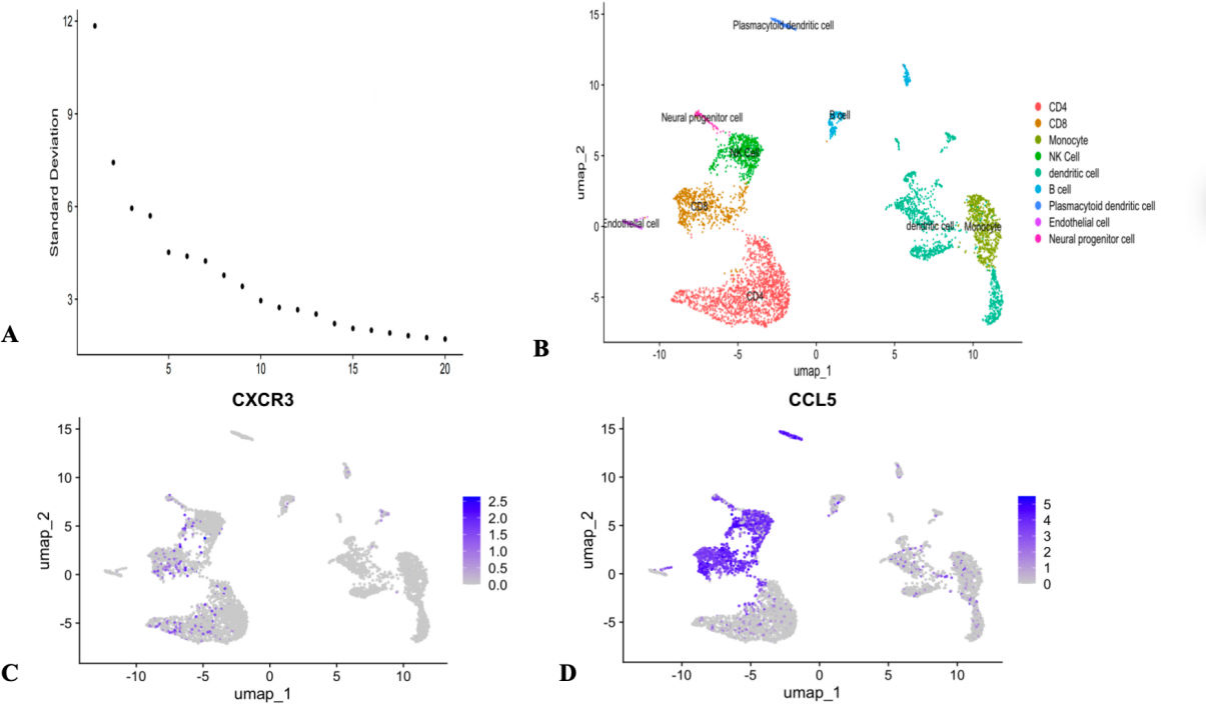
Single-cell analysis results. (**A**) The first 15 cells selected for clustering. (**B**) Cluster annotation for five high HBsAg level patients. (**C**) Expression of CXCR3 in the PBMCs of five high HBsAg level patients.(**D**) Expression of CCL5 in the PBMCs of five high HBsAg level patients.

To further evaluate the role of CXCR3 and CCL5 in the course of chronic hepatitis B and their potential for future treatment, we analyzed single-cell RNA sequencing data from the public database GSE247322, examining the expression of CXCR3 and CCL5 in PBMCs of CHB patients with high viral load. Figure 4A shows the top 15 principal component analysis results of cell clustering, with all cells divided into 9 types (Fig. 4B). The expression of CXCR3 and CCL5 in these 9 identified cell types has been shown in the above results, with higher expression of inflammatory molecules in the IA stage than in IT and LR. Figure 4C visually displays the expression of CXCR3 and CCL5 in various cell types. CXCR3 and CCL5 showed high abundance in natural killer cells (NK cells), CD4, and CD8 T cells. The high expression characteristics of CXCR3 and CCL5 in immune cells provide a theoretical basis for further exploration of their mechanisms and judgment of antiviral treatment.

### Correlation analysis between PMR and oxidative stress, chemokines, and clinical indicators

To further explore the mechanism of FGF21 PMR upregulation in different stages of CHB, we used Spearman rank correlation test to analyze the relationship between FGF21 promoter methylation and FGF21 and its receptor FGFR1, CXCR3, CCL5, Nrf2, and GPX4. Table 3 showed that PMR was negatively correlated with FGF21, FGFR1, CXCR3, and CCL5. We also analyzed the correlation between PMR values and log10[HBV-DNA], HBsAg, HBeAg, ALT, and AST. The results showed that PMR was negatively correlated with log10[HBV-DNA], HBsAg, HBeAg, ALT, and AST.

**TABLE 3:** Correlation analysis between PMR and oxidative stress, chemokines, and clinical indicators

Parameter	PMR (%)	
	*r* value	*P* value
FGF21	−0.2507	<0.0001
FGFR1	−0.1127	0.0426
Nrf2	0.1354	0.0176
CPX4	0.1273	0.0219
CXCR3	−0.1678	0.0024
CCL5	−0.1458	0.0086
HBV-DNA	−0.1416	0.0129
HBsAg	−0.1550	0.0066
HBeAg	−0.2505	<0.0001
ALT	−0.1750	0.0020
AST	−0.1780	0.0017

## DISCUSSION

Chronic hepatitis B (CHB) progresses through distinct immunological phases: immune tolerance (IT), immune activation (IA), low replication (LR), and reactivation (RA). Central to its pathogenesis are virus-driven persistent inflammatory responses and oxidative stress (OS), which interact synergistically to influence disease outcomes. In CHB patients, prolonged viral infection sustains inflammatory infiltration, leading to parenchymal liver necrosis. Without intervention, chronic HBV infection may advance to liver fibrosis, cirrhosis, hepatic failure, and hepatocellular carcinoma, with intrahepatic inflammation being a primary driver of tissue injury (22).

The T cell-mediated immune response to viral antigens is pivotal for both viral clearance and disease pathogenesis in HBV infection. Adaptive immune cells, especially T cells, contribute significantly to chronic liver inflammation and antiviral defense (23). Lebossé et al. (24) demonstrated that chronic HBV infection suppresses innate immunity, an effect exacerbated by high HBsAg levels. These findings imply that reducing HBsAg could potentially restore antiviral immunity. Persistently activated HBV-specific CD8^+^ T cells may exacerbate hepatocyte damage and recruit non-specific T cells in chronic infection (25). The expression of chemokine receptors on peripheral T cells is closely linked to inflammatory status and cytokine equilibrium, highlighting the importance of clarifying key inflammatory and OS pathways in CHB.

FGF21 is increasingly recognized as an early biomarker in various diseases and helps protect organs from damage. Elevated serum FGF21 in patients may represent a compensatory mechanism to counter tissue injury (26). Previous studies indicate that FGF modulates hepatic stellate cell (HSC) activation, apoptosis, and fibrogenesis (27). It suppresses liver inflammation and reduces expression of inflammatory mediators—including components of the NF-κB pathway—at both transcriptional and protein levels. As a key signaling receptor for FGF21, FGFR1 helps regulate immune reactions, metabolic balance, and fibrosis. Wang et al. (28) suggested that the FGF21–FGFR1–heparan sulfate complex may trigger atypical pro-inflammatory signals. In contrast, Yu et al. (29) reported that FGF21 treatment lowers serum AST, ALP, TBIL, and DBIL levels and ameliorates histological liver damage, indicating protection against D-galactosamine-induced injury. Moreover, FGF21 enhances the PGC-1α/Nrf2 pathway to alleviate acetaminophen-induced hepatotoxicity and mitigates D-galactose-induced hepatocyte OS via Nrf2 activation (30). Beyond hepatoprotection, Yan et al. (31) found that FGF21 reduces lipid ROS accumulation, improves mitochondrial function, and inhibits ferroptosis, aiding peripheral nerve repair. Kang et al. (32) showed that FGF21 exerts neuroprotective effects against degenerative changes via NF-κB and AMPK/Akt pathways, underscoring its anti-inflammatory and antioxidant roles.

In liver diseases, Nrf2 and GPX4 are widely studied for their roles in OS response. Nrf2 modulates antioxidant, metabolic, and anti-inflammatory processes and influences cell transformation and antiviral immunity (33). It enhances antioxidant responses and facilitates liver regeneration. Recent evidence indicates that augmenter of liver regeneration (ALR) promotes regenerative processes by diminishing ROS and enabling PI3K-Akt pathway activation (34). Yu et al. (29) demonstrated that FGF21 counteracts OS by activating Nrf2. The FGF21/Nrf2 axis, triggered by AMPK, mitigates apoptosis and inflammation (35), suggesting that FGF21’s protection against OS is partly mediated through inflammatory modulation. Wang et al. (14) observed that rFGF21 significantly reduces pro-inflammatory cytokines (IL-1β, TNF-α, IL-6), suppresses microglial activation, and inhibits NF-κB signaling—effects reversible with FGFR1 inhibition, confirming the role of FGFR1 in FGF21-mediated antidepressant effects.

Besides OS, cytokines significantly contribute to immune dysregulation and tolerance in CHB progression (36). CXCR3 mediates immune cell chemotaxis, particularly for T cells (37). Its upregulation promotes T cell migration to organs, induces cytokine secretion (e.g., IL-6, IFN-γ), and exacerbates inflammation (38). CXCR3 knockdown suppresses TLR4/MyD88, BIRC, and COL1A1 expression, inhibits proliferation and migration, and promotes apoptosis, thereby attenuating cirrhosis-to-carcinoma transition (39). CXCR3 deficiency also ameliorates ANIT-induced bile acid metabolic dysregulation, reducing recruitment of IFN-γ^+^ and IL-4^+^ NK cells and suppressing ER stress (19). Similarly, CCL5 facilitates leukocyte chemotaxis and activation (15). It signals through CCR1, CCR3, and CCR5 receptors, activating transcription factor 3, NF-κB, and MAPK pathways. Elevated serum CCL5 in CHB- and HBV-related cirrhosis correlates with disease severity (40, 41), supporting its utility as a biomarker for immune status assessment in CHB.

DNA methylation, a key epigenetic mechanism, is influenced by inflammation and OS. Catalyzed by DNMTs, DNA hypomethylation can destabilize oncogenes and promote carcinogenesis, whereas promoter hypermethylation represses gene transcription (42). Li et al. (43) linked IGFBP7 promoter methylation to liver metastasis risk in colorectal cancer via OS and DNMT expression. Wang et al. (44) observed OS-induced MDM2 hypomethylation in liver cancer, and Li et al. (45) proposed SOCS1 methylation as a predictor of glucocorticoid response in acute-on-chronic hepatitis B liver failure. OS-driven epigenetic changes likely contribute to CHB development. Su et al. (46) showed that GPX4 downregulation in CHB patients’ PBMCs results from promoter hypermethylation, most pronounced in the immune tolerance phase, suggesting a role in viral persistence. Our previous study (47) also found variable FGF21 promoter methylation across HBeAg serological statuses.

**Fig 5:**
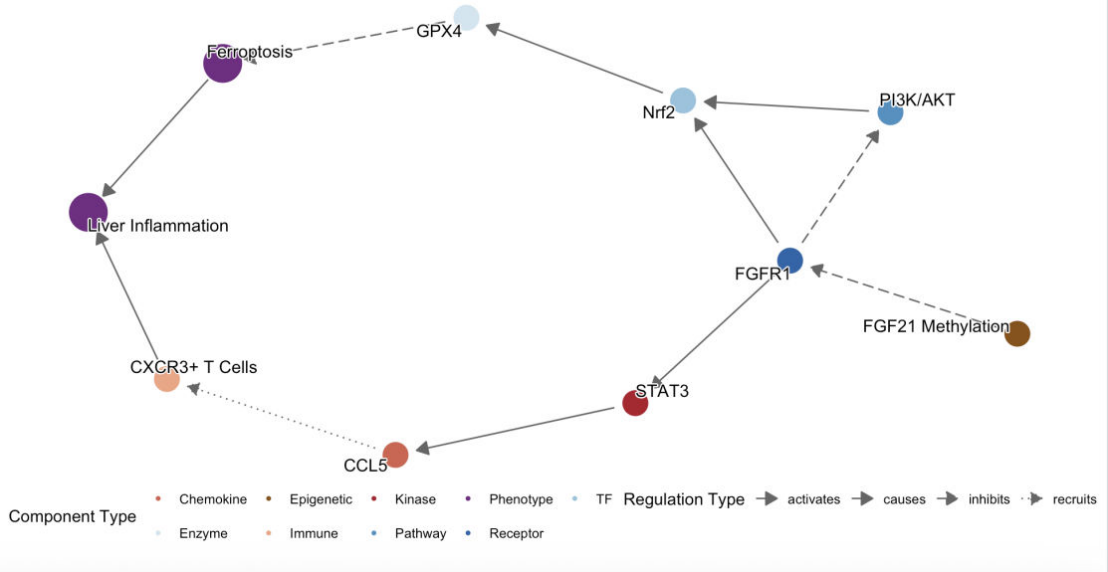
FGF21 methylation regulates inflammation-oxidation network.

To explore FGF21 promoter methylation in CHB immune stages and its interplay with immune inflammation and OS, we analyzed clinical samples. We found that the FGF21 mRNA levels were significantly lower in the IT, LR (*P* < 0.0001), and HC groups compared to the IA group, and also lower in the IT, LR, and HC groups compared to the RA group. The relative mRNA levels of FGFR1 were significantly lower in the IT, LR, and RA groups compared to the IA group. The PMR levels of FGF21 were significantly higher in the IT, LR, and HC groups compared to the IA group, and higher in the LR and HC groups compared to the IT group. The relative mRNA levels of CXCR3 were significantly lower in the IT, LR, and HC groups compared to the IA group, and also lower in the IT, LR, and HC groups compared to the RA group. The relative mRNA levels of CCL5 were significantly lower in the IT, LR, and HC groups compared to the IA group, and also lower in the IT, LR, and HC groups compared to the RA group. The relative mRNA levels of Nrf2 were significantly lower in the IT and HC groups compared to the IA group and also lower in the IT and HC groups compared to the RA group. The relative mRNA levels of GPX4 were significantly lower in the IT, IA, and LR groups compared to the RA group and also lower in the IT and LR groups compared to the HC group. The results indicate that the elevated expression of FGF21 in PBMCs of CHB patients is caused by demethylation, and the expression level is most significantly elevated during the immune activation phase, suggesting that FGF21 demethylation may be involved in the formation of the immune activation phase in CHB patients, leading to HBV OS and the occurrence of pro-inflammatory factor storms. Additionally, as the receptor of FGF21, the changes in FGFR1 are consistent with FGF21 mRNA, confirming the synergistic effect of the two in previous studies. At the same time, during the immune tolerance phase and immune activation phase with high HBsAg, we also found high expression of CXCR3 and CCL, which is consistent with previous single-cell sequencing studies, suggesting that CXCR3 and CCL5 promote T cell infiltration, and the upregulation of chemokine receptor expression in immune cells under high HBsAg status may be one of the mechanisms of viral escape. In the reactivation phase with decreased viral load, FGF21, FGFR1, CXCR3, and CCL5 are elevated again, which may be related to immune reconstruction damage and the occurrence of epigenetic-oxidative inflammatory storms. The expression levels of Nrf2 and GPX4 are most significantly reduced during the immune tolerance phase, suggesting that this phenomenon may be involved in the formation of immune tolerance in CHB patients, leading to chronic HBV infection, which is consistent with the findings of Su et al. In the reactivation phase, Nrf2 and GPX4 are elevated again, and viral reactivation causes acute liver injury and exacerbates fibrosis, with compensatory high expression of Nrf2 and GPX4, and acute OS promotes transient activation of Nrf2 and GPX4, but their functions may be dysregulated due to continuous ROS exposure. To further illustrate the relationship between FGF21 promoter methylation and OS and immune inflammatory factors, we conducted a correlation analysis, which showed that PMR is negatively correlated with FGF21, FGFR1, CXCR3, and CCL5, and positively correlated with Nrf2 and GPX4, indicating that high levels of ROS promote Nrf2 nuclear translocation by inhibiting KEAP1, binding to the antioxidant response element (ARE) in the FGF21 promoter, and enhancing its transcription. OS and inflammatory factors synergistically activate the MAPK/ERK and PI3K/AKT pathways, further amplifying the FGF21 expression signal. Virus-mediated DNA methylation inhibition, HBx forms a complex with DNMTs, inhibiting their methylation activity, leading to FGF21 promoter hypomethylation, and HBx reduces the supply of methyltransferases by inhibiting DNMT transcription. These findings are preliminary and require further validation in larger cohorts and functional experiments to determine their causal relationship and true pathophysiological contribution. Additionally, through correlation analysis with viral load and liver function, it was found that the FGF21 promoter methylation level is negatively correlated with log10[HBV-DNA], HBsAg, HBeAg, ALT, and AST, which is consistent with the high expression of CXCR3 and CCL5 chemokines caused by high viral load in single-cell results, inflammation and immune activation, and aggravated liver damage. This study identifies FGF21 promoter methylation as an epigenetic nexus linking immune inflammation and OS in CHB. We propose a methylation–chemokine–OS interaction network (Fig. 5): FGF21, a hepatic metabolic regulator, upon demethylation, binds FGFR1 to activate antioxidant (Nrf2/GPX4) and anti-inflammatory (via STAT3-CCL5 inhibition) pathways, bridging OS and inflammation. Nrf2 upregulates GPX4 to maintain redox homeostasis and inhibit ferroptosis. Meanwhile, CCL5 and CXCR3 recruit CD4^+^ and CD8^+^ T cells, activating NF-κB and releasing TNF-α and IL-6, worsening hepatocyte necrosis. In high-viral-load patients, OS and immune inflammation drive epigenetic remodeling, causing FGF21 hypomethylation and high expression—a dual response that may both adaptively counter viral damage and facilitate immune escape.

Our study has limitations. First, the single-center design and the small sample size may lead to selection bias. Moreover, there are issues such as the absence of data on body mass index, alcohol consumption, and differences in age and gender, which we have not thoroughly discussed. In future studies, we will incorporate more comprehensive variables for in-depth analysis. Second, while correlations between FGF21 and cytokines were assessed, causal regulatory mechanisms remain unclear and require validation via cellular and animal models. Future work integrating single-cell multi-omics and dynamic metabolic profiling could enable personalized molecular network analysis, advancing CHB management from broad antivirals to precision immunomodulation.

### Conclusion

CHB patients show FGF21 promoter hypomethylation, most prominently in the immune activation phase. FGF21 methylation correlates with OS and immune-inflammatory cytokine expression, suggesting a role in antiviral treatment response and seroconversion. Targeting this epigenetic node may disrupt the OS–inflammation cycle, offering a novel strategy for phase-specific precision therapy. However, the exact role of FGF21 in CHB pathogenesis and its therapeutic potential require further investigation.

## Data Availability

The data presented in this study are included in the article. Further inquiries can be directed to the corresponding author.
